# The burden of allergic rhinitis (AR) in Canada: perspectives of physicians and patients

**DOI:** 10.1186/1710-1492-8-7

**Published:** 2012-06-01

**Authors:** Paul K Keith, Martin Desrosiers, Tina Laister, R Robert Schellenberg, Susan Waserman

**Affiliations:** 1 McMaster University, Hamilton, ON, Canada; 2 Hôtel-Dieu de Montréal, Montreal, QC, Canada; 3 Nycomed: A Takeda Company, Oakville, ON, Canada; 4 Pacific Lung Health Centre, St. Paul's Hospital, Vancouver, BC, Canada

**Keywords:** Rhinitis, Allergic, Anti-allergic agents, Administration, Intranasal, Quality of life

## Abstract

**Background:**

Allergic rhinitis (AR) is a common problem and we sought to examine the burden of disease and its management in Canada from the perspectives of patients and physicians.

**Methods:**

Two parallel, Canadawide structured telephone interviews surveyed 1,001 AR patients and 160 physicians in July 2006.

**Results:**

44% of patients had experienced nasal symptoms unrelated to a cold and 20% had a physician diagnosis of AR. At screening 27% reported asthma, 15% chronic or recurrent sinusitis and 5% nasal polyps. With attacks nasal congestion and runny nose were the most bothersome symptoms. Other problems experienced were fatigue (46%), poor concentration (32%), and reduced productivity (23%). Most (77%) had not seen a physician in the past year. Physicians estimated they prescribed intranasal cortico steroids (INCS) to most AR patients (77%) consistent with guidelines but only 19% of patients had used one in the last month. Only 48% of patients were very satisfied with their current INCS. 41% of AR patients reported discontinuing their INCS with the most common reason being a perceived lack of long-lasting symptom relief (44%). 52% of patients felt that their current INCS lost effectiveness over 24 h. The most common INCS side effects included dripping down the throat, bad taste, and dryness. Most AR patients reported lifestyle limitations despite treatment (66%). 61% of patients felt that their symptoms were only somewhat controlled or poorly/not controlled during their worst month in the past year.

**Conclusions:**

AR symptoms are common and many patients experience inadequate control. Physicians report they commonly prescribe intranasal corticosteroids, but patient’s perceived loss of efficacy and side effects lead to their discontinuation. Persistent relief of allergic rhinitis symptoms remains a major unmet need. Better treatments and education are required.

## Background

Allergic rhinitis (AR) is an inflammatory disease of the nasal mucous membranes [1,2]. Allergen exposure of allergic individuals results in an IgE-mediated inflammatory response, which is manifested clinically as rhinorrhea, nasal congestion, postnasal drainage, nasal itching, sneezing, and itchy or watery eyes [1,2]. AR is common and previously estimated to affect approximately 20–25% of Canadians [3]. The prevalence of AR is increasing worldwide, a trend that has been attributed to a variety of factors such as changing global climate conditions, improvements in hygiene, changes in diet, and increased obesity [1,4,5]. Rhinitis whether atopic or nonatopic is a risk factor for the development of asthma. The more persistent and severe the rhinitis, the more likely one may go on to develop asthma.

Allergen avoidance and pharmacotherapy are the cornerstones of AR management [6,7]. Pharmacotherapy is individualized to the patient based on type of symptoms, their duration and severity, comorbidities, response to prior treatment, and patient preference [1,6]. Classes of drugs used to treat AR include antihistamines, corticosteroids, mast cell stabilizers, decongestants, nasal anticholinergics, and leukotriene-receptor antagonists [1,6-8]. Guidelines recommend INCS as treatment for patients with moderate to severe AR and/or persistent symptoms [1,7]. Extensive clinical evidence indicates that INCS provide greater relief of AR symptoms than antihistamines [9,10]. The European position paper on rhinosinusitis and nasal polyps 2007 (EPOS) guideline recommends INCS if there is a moderate degree of nasal congestion even if allergy skin tests are negative.

The perspectives of patients and physicians on the burden of disease for AR and its management have been described for the United States [11-13]. However, similar data for Canada do not exist. (Pub Med search November 17, 2011: “allergic rhinitis” and “Canada”) This report highlights findings from *Allergies in Canada,* a study consisting of two parallel, nationwide surveys of 3,671 adults with further questioning of 1,001 adult nasal allergy sufferers as well as 160 physicians in Canada. The objectives of the *Allergies in Canada* survey were to examine the breadth of symptoms, disease burden, and current management of AR in Canada from the perspective of physicians and patients.

## Method

### Sample

A cross-sectional sample of adults patients in Canada was obtained through random-digit dialing, telephone screening of households nationwide. Cooperative individuals were eligible to participate in the patient survey if they were ≥18 years old. In screening, a random subset of cooperative individuals ≥18 years old were asked if they had other common upper and lower respiratory problems including asthma, sinusitis and nasal polyps since the Canadian prevalence of these conditions is unknown.

Those cooperative individuals ≥18 years old and suffering from AR as defined by:

· Receipt of a physician diagnosis of AR, nasal allergies, or “hay fever”

OR

· Use of prescription or over-the-counter medications to treat self-diagnosed nasal congestion or symptoms (unrelated to cold or flu).

A representative sample of Canadian physicians who treat AR was obtained by random screening of a Canadian physician database comprised of active clinicians who have experience treating with INCS.

The goal was to obtain a sample of 1,000 patients. The maximum expected sampling error for a simple random sample of 1,001 (the patient sample) is ±1.9 percentage points at the 95% confidence level. The maximum expected sampling error for a simple random sample of 100 (the general practice/family medicine sample) is ±4.8 percentage points at the 95% confidence level.

### Survey questionnaires

Previous patient and physician questionnaires used in the *Allergies in America* study [13,14] were reviewed, modified, and approved by a panel of Canadian AR experts. The surveys contained questions about AR diagnosis, symptoms, comorbidities, quality of life impact, and treatment. Attitudes toward the disease, expectations for treatment, and educational needs were also examined. The majority of responses were elicited in a directed manner, i.e., responses could be yes/no or one of a discrete number of prompted answers. The patient survey contained 73 questions; the physician survey contained 55 questions. Patient and physician respondents completed the surveys in structured telephone interviews conducted in July 2006. All interviews were conducted by Leon Wahler and Live Contact Inc. The interview team was comprised of 11 individuals.

Patient-reported data corresponds to the actual percentages of patients providing a particular response. Physician-reported data on the characteristics of their own AR patient population corresponds to the averages of all physician responses.

An analysis was performed of symptom control in the worst month in the subgroups of patients using over-the-counter (OTC) vs prescription medication.

LiveContact, the research provider for this study, guaranteed privacy and confidentiality for all study respondents and conducted the survey and data collection according to Canadian privacy legislation (PIPEDA) and marketing research guidelines established by the Marketing Research and Intelligence Association (MRIA) in Canada. Based on the anonymous nature of the survey, the lack of intervention and the implied consent by completing the survey by the subjects our research ethics board at the time felt this was a quality assurance project and deemed it did not require formal submission.

## Results

### Profile of respondents to the patient survey

30,987 telephone calls to households were made. Among 5,348 respondents to the initial telephone contact, there were 3,671 respondents who were ≥18 years old and cooperative (willing to discuss medical history and answer screening survey questions). Of these, 44% (1,610/3,671) had experienced nasal symptoms and 20% (734/3,671) had been formally diagnosed with AR, nasal allergies, or hay fever by a physician. Among the subset of cooperative respondents asked further about specific comorbid conditions, 27% reported asthma, 17% reported having been diagnosed with chronic or recurrent sinusitis (n = 1,595 respondents queried) and 5% had nasal polyps (n = 1,591 respondents queried).

1,001 respondents reported that they had used prescription or OTC medication to treat nasal symptoms OR had a diagnosis of AR and were willing to proceed with the remainder of the survey. These 1,001 individuals affected with AR completed the survey questionnaire. Unless otherwise specified, this cohort of 1,001 composes the “patients” whose perspectives are described in this report (see Table 1). Although not all affected individuals were under active medical care, the term “patients” is used herein for simplicity. The average interview time for patients was 28 minutes.

**Table 1:** Patient population completing the AR survey

**Patient population description**	**Number of patients**
Initial contacts	30,987
Respondents to initial telephone contact	5,348
Cooperative adult respondents	3,671
Cooperative adult respondents who had experienced nasal allergy symptoms	1,610
Respondents who had used prescription or OTC medication to treat their nasal allergy symptoms	1,001

81% of the 1,001 patients surveyed had sought medical attention for their nasal symptoms at some point and 63% of these patients had been diagnosed with nasal allergies, AR, or hay fever by a physician.

### Profile of respondents to the physician survey

3,392 telephone calls to physicians were made and 1,258 physicians were asked to participate in the survey. 160 physicians completed the physician questionnaire including 100 in general practice/family medicine, 30 allergists, and 30 otolaryngologists. The average interview time for physicians was 25 minutes.

General practice/family medicine physicians reported that on average 18% of patients in their practices suffer from AR. Among allergists and otolaryngologists the average percentage of patients in the practice that suffer from AR is 60% and 23%, respectively.

### Common comorbid conditions

**Figure 1:**
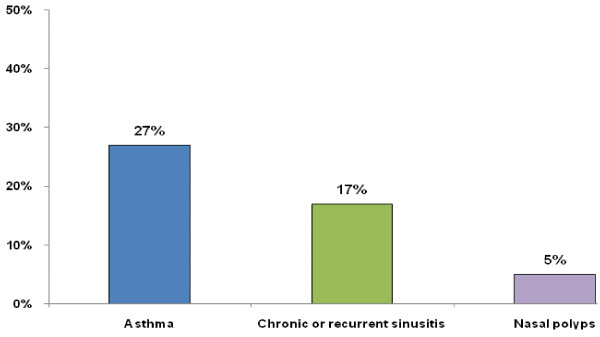
Percentage of AR patients surveyed reporting a physician diagnosis for selected comorbid conditions.

27% of 1,001 AR patients reported having a physician diagnosis of asthma, 17% reported physician-diagnosed chronic or recurrent sinusitis, and 5% reported physician-diagnosed nasal polyps (see Figure 1).

**Figure 2:**
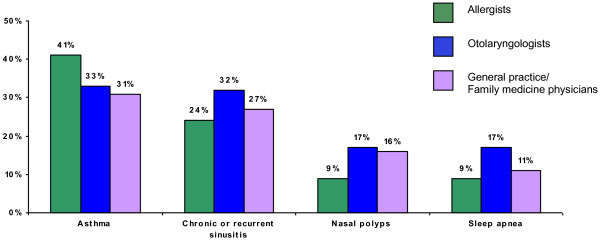
Physician estimates of the percentage of AR patients who suffer from selected comorbid conditions (average by physician specialty).

When asked to estimate the percentage of AR patients in their practices with selected comorbid conditions, physicians reported an average prevalence of 33% for asthma, 28% for chronic or recurrent sinusitis, 15% for nasal polyps, and 12% for sleep apnea (see Figure 2).

90% of physicians felt that poor control of nasal allergy symptoms could make asthma worse and 92% felt that nasal allergies could cause sinus infections.

### Symptom burden

**Figure 3:**
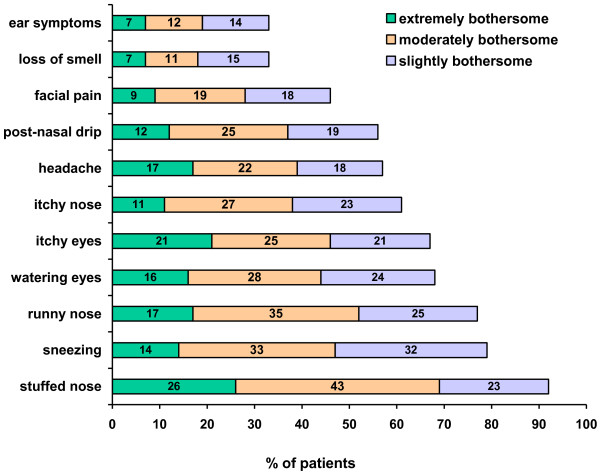
Nasal allergy symptoms most commonly reported by patients to be extremely or moderately bothersome.

The nasal allergy symptom that was most often reported by patients to be moderately or extremely bothersome was stuffed nose (69% of patients). The next most common symptoms reported to be extremely or moderately bothersome were runny nose (52%) and sneezing (47%) (see Figure 3). Two-thirds of patients experienced stuffed nose every day (35%) or a few days per week (32%) during their worst month in the past year. Over half of patients experienced sneezing (59%) and runny nose (53%) at least a few days per week in their worst month.

Both patients and physicians reported high rates of perennial nasal symptoms. Among the patients surveyed, 49% reported year-round symptoms. Physicians reported that 60% of their patients have perennial nasal symptoms.

Physicians recognize a significant burden of disease from AR symptoms in their patients. Overall, they reported that 29% of their AR patients suffer from severe symptoms and 42% from moderate symptoms. Allergists’ estimate of the percentage of their patients with severe symptoms (35%) while otolaryngologists (25%) and general practice/family medicine physicians (29%) felt that the percentage was less.

**Figure 4:**
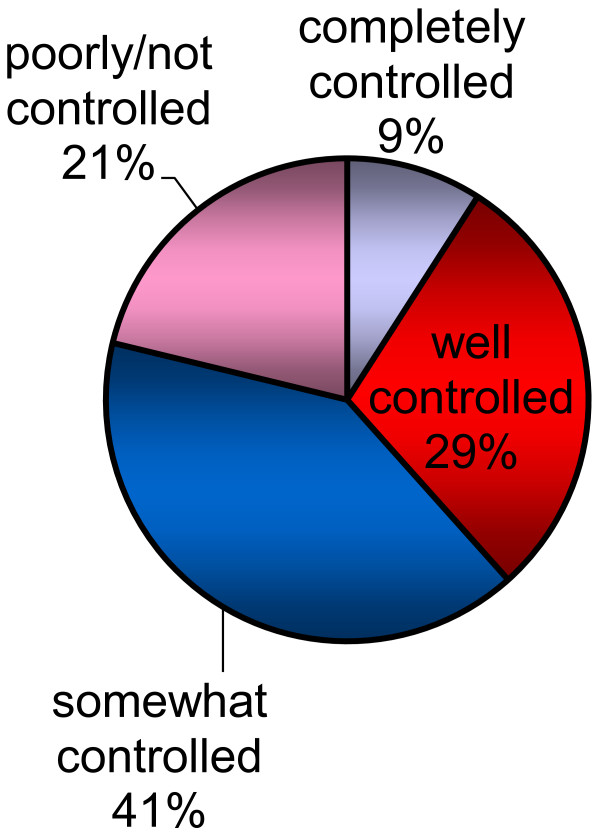
Allergic rhinitis symptom control in worst month.

AR symptom control was felt to be suboptimal by patients. 61% of patients felt that their symptoms were only somewhat controlled or poorly/not controlled during their worst month in the past year. Among those patients, 60% reported that their symptoms were only somewhat controlled or poorly/not controlled in the past month (see Figure 4).

### Impact of AR on daily life

**Figure 5:**
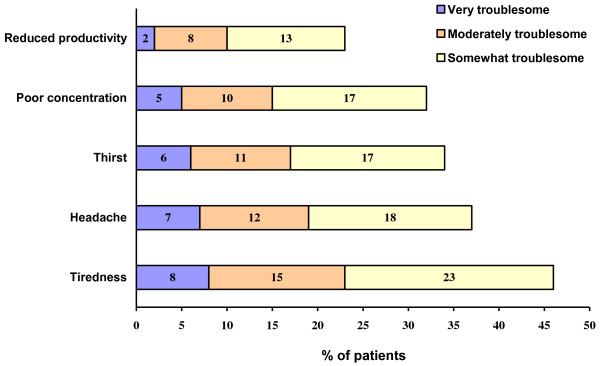
Degree of affliction of nasal allergy symptoms from the perspective of patients.

72% of patients indicated that during allergy season their AR symptoms adversely impacted their daily lives. The most troublesome problems reported by patients were fatigue (46%) and headache (37%). Poor concentration and reduced productivity are also common troublesome problems (see Figure 5).

### Diagnosis and treatment of AR

Regarding diagnosis of AR, allergy skin tests are the most commonly used diagnostic test. 80% of allergists reported always using skin tests compared with 17% of otolaryngologists (either in their own practice or by referral) and 8% of general practice/family practice physicians (either in their own practice or by referral). Most physicians (73%) rarely or never use blood tests (which measure serum specific IgE to inhalant allergens for diagnosis of AR [1]). Of patients who had sought medical attention for nasal allergy symptoms, 44% had skin testing and 13% had had blood tests.

**Figure 6:**
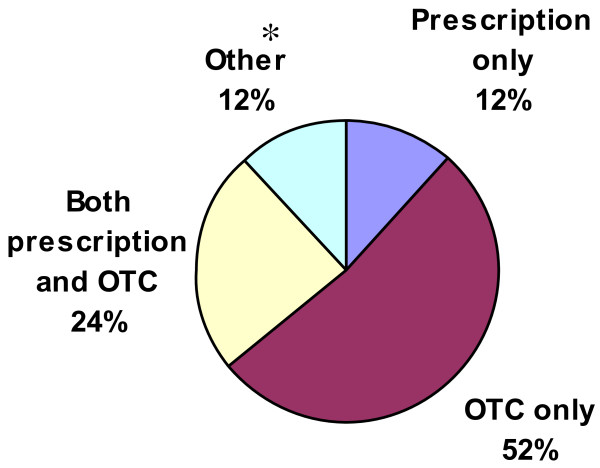
**Medications used for the current management of AR symptoms as reported by patients (% of patients).** * The “Other” category includes patients who reported that they used to take prescription medication, used to take OTC medication, used to take both prescription and OTC medication, or did not know/did not have an answer.

52% of patients reported using only non-prescription, OTC products to control their allergy symptoms and 36% reported using prescription medication (see Figure 6). Based on physicians’ estimates, prescription medication for AR is typically INCS—on average, 78% of allergists’ patients, 83% of otolaryngologists’ patients, and 75% of general practice/family medicine physicians’ AR patients were being prescribed INCS. Despite reported high rates of INCS prescribing by physicians, only 19% of patients reported INCS use in the past month. However, only 23% of the patients surveyed had seen a physician about AR symptoms in the past 12 months.

Of patients who had seen a physician for their nasal allergies (81% of patients surveyed), 22% had been treated with immunotherapy at some point in time. Overall, physicians estimated 15% of their patients (19% of allergists’ patients, 11% of otolaryngologists’ patients, and 14% of general practice/family medicine physicians’ patients) were currently or ever had received immunotherapy.

Among patients who had seen a physician for their nasal allergies, 58% reported that their physician had demonstrated how to use a nasal spray device. In comparison, when physicians were asked 90% of allergists, 87% of otolaryngologists, and 75% of general practice/family medicine physicians estimated that they demonstrate INCS spray technique once a year or when they prescribe a product.

### AR treatment experience

When symptom control in the worst month was compared between the subgroups of patients using OTC vs prescription medication, there was no apparent difference between subgroups. The use of prescription medication such as INCS was not associated with higher rates of completely or well controlled symptoms in the worst month.

**Figure 7:**
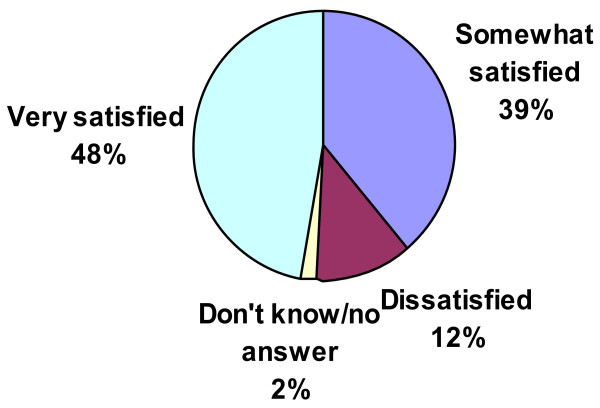
Patients’ degree of satisfaction with current INCS (% of patients).

**Figure 8:**
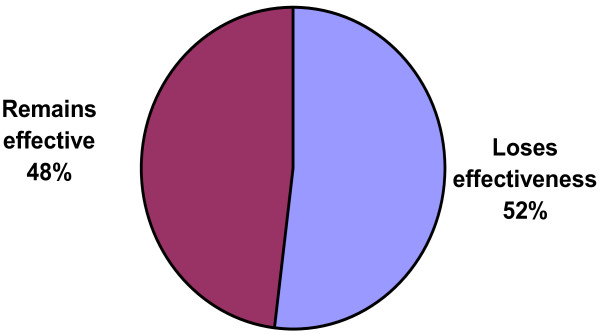
Ability of current INCS to maintain effectiveness over a 24-hour period from patients’ perspectives (% of patients).

Among patients using INCS to control AR, only 48% reported being very satisfied with their current INCS, whereas 12% reported being dissatisfied (see Figure 7). In contrast, physicians estimated that only 2% of their patients were dissatisfied with their INCS. Regarding symptom control, 35% of patients reported that their current INCS relieved only some or none of their allergy symptoms, whereas 85% of physicians reported that INCS generally relieve most or all allergy symptoms in their patients. 52% of patients perceived that their current INCS loses effectiveness over the course of a 24-hour period (see Figure 8); only 35% of physicians shared this perception.

**Figure 9:**
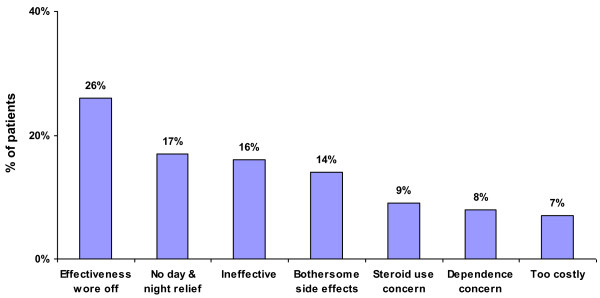
Percentage of patients reporting a particular reason for discontinuation of a prescribed nasal allergy medication.

The rates of discontinuation of prescription medication reported by patients and physicians were 41% and 33%, respectively. As shown in Figure 9, the two most common reasons patients report for INCS discontinuation relate to failure of treatment to provide long-lasting symptom relief, including diminished effectiveness (26%) and loss of effectiveness over a 24-hour period (17%).

**Figure 10:**
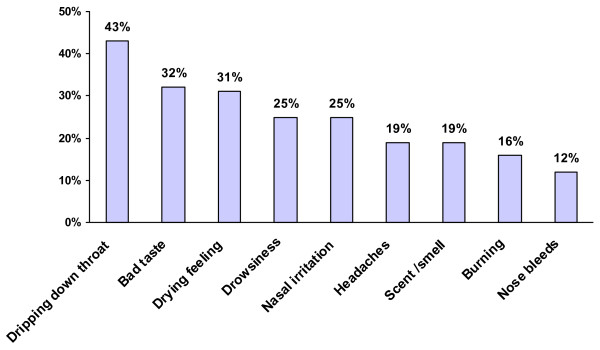
Side effects of INCS reported by patients (% of patients).

Other reasons for INCS discontinuation were bothersome side effects, cited by 14% of patients. The side effects of INCS most commonly reported by patients were dripping down the throat (43%), bad taste (32%), and dryness (31%) as shown in Figure 10.

### Expectations for AR treatment

Patients’ and physicians’ expectations for onset of action, duration of effect, and overall effectiveness of INCS were markedly different, with patients generally expecting a faster onset of action and greater degree of symptom relief (see Table 2). A significant percentage of patients were not informed about what to expect with regard to INCS onset of action (41%) and duration of effect (42%). Most physicians (77%) expect INCS to have duration of effect of 24 hours or more.

**Table 2:** Expectations of intranasal corticosteroid treatment (% of respondents)

	**Patients**	**Physicians**
**Onset of action**	<24 hours: 52%	<24 hours: 6%
**Duration of action**	≥24 hours: 15%	≥24 hours: 77%
**Definition of treatment success**	All symptoms relieved: 41%	All symptoms relieved: 6%

**Figure 11:**
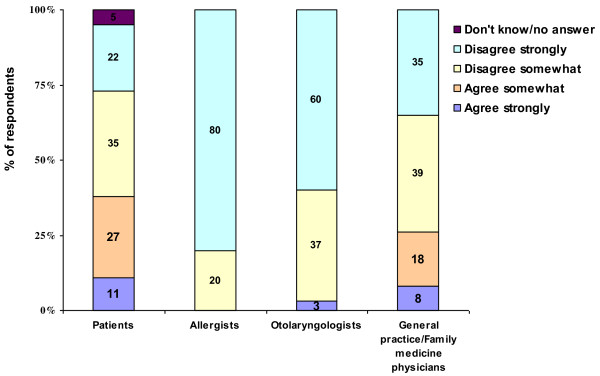
Patients’ and physicians’ attitudes toward the statement “there are no truly effective treatments for nasal allergies” (% of respondents).

Patients and physicians differed in their attitudes regarding the prevention and treatment of AR symptoms. While 100% of allergists, 90% of otolaryngologists, and 83% of general practice/family medicine physicians felt that frequent AR symptoms can generally be prevented, only 64% of patients felt that symptoms can be prevented. 66% of AR patients and 71% of physicians agreed with the statement that even with proper treatment nasal allergies usually cause some lifestyle limitations. 38% of patients agreed with the statement that there are no truly effective treatments for nasal allergies, while only 26% of general practice/family medicine physicians, 3% of otolaryngologists, and 0 allergists agreed (see Figure 11).

### Knowledge about AR and educational needs

Physicians cited a variety of professional guidelines for the management of AR without prompting, with those from the Allergic Rhinitis and its Impact on Asthma Workshop Group (ARIA; cited by 33% of allergists, 10% of otolaryngologists, and 0 general practice/family medicine physicians) [1,15], the Canadian Society of Allergy and Clinical Immunology (CSACI; cited by 17% of allergists, 0 otolaryngologists, and 2% of general medicine/family medicine physicians) [16], and the American Academy of Allergy Asthma and Immunology (AAAAI; cited by 17% of allergists, 0 otolaryngologists, and 0 general medicine/family medicine physicians) [8] most commonly mentioned. With prompting, 70% of allergists, 33% of otolaryngologists, and 7% of general medicine/family medicine physicians indicated that they had heard of the ARIA guidelines. Most physicians (96%) and patients (91%) perceived that there is a strong or moderate need for better education of nasal allergy patients about their condition.

## Discussion

The *Allergies in Canada* study included the largest patient and physician surveys of AR and common comorbid conditions conducted to date in Canada.(Pub Med search November 17, 2011: “allergic rhinitis” and “Canada”) These data confirm and extend findings reported for AR patient populations in other countries with regard to burden of disease [11-13].

As with all telephone surveys, certain limitations in interpretation exist. Surveys in general may be associated with bias in that the survey sample may not always be representative of the population. Certain subsets of individuals may be more likely to be unavailable to participate in a telephone survey. The criteria for AR patients in this study allowed for inclusion of AR sufferers who had not received a physician diagnosis of AR, provided they had symptoms consistent with AR that they self-treated with medication. Patients were not subjected to physical examination or skin tests. This may have permitted inclusion of some patients who did not have AR, for example, it is possible that some patients with nonallergic rhinitis could have been included. If the sample size of this study had been larger, more inferences might have been drawn regarding the impact of demographic factors on AR experiences and attitudes. The surveys were conducted in July 2006 and some currently available AR medications (e.g., the INCS, ciclesonide and fluticasone furoate) were not available at that time.

In this study, the prevalence of physician-diagnosed AR in Canadian adults in the general population and in the patient population seen in general practice/family practice paralleled the 20–25% prevalence of AR previously reported [3]. The prevalence of self-reported nasal allergy symptoms among all cooperative respondents was 44%. This larger percentage may be due to the fact that the respondent population may have included a number of subjects with nonallergic rhinitis or that a substantial number of AR sufferers are either not seeking medical attention for their condition or are not diagnosed.

Nasal congestion and runny nose were identified as the most bothersome symptoms of AR and were reported to occur frequently. This parallels findings of a recent survey of adult AR patients in the United States [13]. Similarly, nasal congestion was moderate to severe in 80% of patients with uncontrolled rhinitis seen by a family practitioner in a recent study of seasonal AR in Canada [17]. Control of nasal congestion and runny nose should be a primary goal of AR therapy.

Most patients reported an adverse impact of nasal allergy symptoms on their daily life, a problem not always appreciated by physicians and patients. Major problems due to allergy symptoms cited by patients included fatigue, headache, poor concentration, and reduced productivity. These findings are consistent with the high rates of sleep disorders, daytime fatigue, memory impairment, and reduced work productivity in AR patients described in previous studies [18-20]. Other studies have documented the adverse effects of nasal allergies on patients’ psychological well-being and social functioning [12,13,21]. Effective symptom control is essential to preserving quality of life in AR patients [22].

This study provided a comprehensive description of current management of AR in Canada. Results show that more than one-third (37%) of nasal allergy symptom sufferers who had sought medical care for their symptoms had not received a physician diagnosis of AR. It was not determined whether these patients received alternative diagnoses (e.g., nonallergic rhinitis) or went undiagnosed. However, the prevalence of nonallergic, noninfectious rhinitis in the general population is estimated to be low (2-4%) compared with AR (approximately 20%);therefore, this would not be expected to contribute substantially to the observed lack of AR diagnosis [6,15]. The data suggest there might be substantial underdiagnosis of AR in Canada. Prompt AR diagnosis is important to achieve optimal symptom management.

Approximately half of patients surveyed were treating AR with OTC medication only and 36% were using prescription medication. The prescription medication usage rate parallels that seen in a recent study of seasonal AR in Canada in which 30% of subjects reported currently using prescription medication, specifically INCS, to control AR [17]. Among patients treated by physicians, INCS use is estimated to be high (77%) consistent with published guidelines for the treatment of moderate-to-severe persistent AR [1,7,15].

Only a small subset (15%) of patients were estimated by physicians to be receiving allergy shots, consistent with guidelines that recommend immunotherapy be reserved for more severe disease and where symptoms are inadequately managed by maximum pharmacologic therapy [6,8]. Although from the patient survey 19% of patients had received allergen immunotherapy in the past. Only 5% of patients with uncontrolled rhinitis seen by a family practitioner in the recent study of seasonal AR in Canada were currently receiving immunotherapy [17]. Although patients surveyed stated they had blood tests to make the diagnosis of allergic rhinitis it is not necessarily true that they had had specific serum IgE testing as physicians surveyed generally did not use this test to make the diagnosis. They may have had screening blood work and the diagnosis may have been made on history alone.

Guidelines recommend that patients be instructed in the use of intranasal corticosteroid sprays to minimize the occurrence of side effects [8,15]. Demonstration rates reported by physicians were relatively high (80% of physicians surveyed estimated that they demonstrate INCS spray technique once a year or when they prescribe a product), however a substantial percentage of patients (42%) reported no nasal spray demonstration. Certain patients may need nasal spray use demonstrated more frequently to ensure that they retain the information, and to increase compliance and minimize side effects.

Patients are not completely satisfied with INCS. Lack of long-lasting symptom relief is a concern and the most common reason for treatment discontinuation. Poor tolerability is the second most common reason for treatment discontinuation. Consistent with the findings of other AR studies, dripping down the throat, bad taste, and dryness are among the most commonly reported side effects [13]. Drowsiness, which was reported by 25% of patients in this study, is not commonly associated with INCS and may perhaps be attributable to concomitant OTC medication in some patients, such as sedating antihistamines, and/or to AR-related fatigue. The majority of physicians (85%) reported that INCS generally relieve most or all allergy symptoms. In contrast to physicians’ perceptions, 35% of patients reported that their current INCS relieve only some or no allergy symptoms. This might be attributed to their as needed use. Patients generally expected a faster onset of action and greater degree of symptom relief associated with INCS than physicians. Physicians generally expected a longer duration of INCS action than patients. Although INCS generally have a measurable benefit within hours, the maximal effect may require up to 2 weeks [1]. The study results suggest that physicians generally do not acknowledge the immediate benefit of INCS. Patients who are uninformed about the time to maximal INCS effect may be more likely to use INCS *PRN* (as needed), regardless of the prescribed dosing regimen.

Most patients and physicians perceived that AR causes lifestyle limitations even when properly treated. More than twice as many patients (38%) as physicians (17%) do not believe that truly effective AR treatment exists. Patients’ lack of confidence in the existence of effective treatment is perhaps attributable to their own experiences. Most patients (61%) reported that their symptoms were only somewhat controlled, poorly controlled, or not controlled during the worst month in the past year and few patients (23%) reported seeing a physician for AR management in the past year.

The gaps identified between patients and physicians with respect to expectations for treatment and treatment outcomes suggest areas that might benefit from better communication. When initiating treatment, patients should be instructed on what to expect regarding a response. Patients should be encouraged to report any suboptimal treatment response so that appropriate intervention can be made.

Comorbid conditions are often overlooked yet contribute significantly to the burden of AR. Asthma, sinusitis, nasal polyposis, and sleep apnea were reported in a significant number of patients in this study.) The contribution of rhinitis whether allergic or nonallergic to the development of asthma, sinusitis, and sleep apnea has been documented [23-25]. In addition, an association between AR and nasal polyposis has been presumed although not conclusively demonstrated [23,26]. It was encouraging that the links between AR and airway diseases were widely recognized by the physicians in this study. Assessment for comorbid conditions should be an integral part of care of AR patients.

Unprompted awareness of AR management guidelines was relatively low among physicians, particularly among those in general practice/family medicine, suggesting a need for more effective guidelines dissemination. Treatment consistent with expert consensus guidelines has been shown to result in significantly better patient outcomes than nonstandardized treatment [27]. Better education about AR was recognized as a major unmet need for patients in this study. The discrepancy between patients’ and physicians’ expectations for INCS found in this study suggests that AR treatment is one of the areas in which better patient education is warranted.

It is hoped that the questionnaires used in this study provide the basis for development of validated, standardized, comprehensive AR questionnaires. Validated, standardized, comprehensive AR questionnaires did not exist before the development of the *Allergies in America* survey questionnaires [13], which formed the basis for the *Allergies in Canada* survey questionnaires. Content validity of the survey questionnaires was determined by a panel of Canadian AR experts, however, the questionnaires were not fully validated. The survey questionnaires were generally consistent with those used to study particular aspects of AR (symptom burden, activity impairment) in accepted health and rhinitis surveys [28-30].

## Conclusions

Many AR patients in Canada experience symptoms that could be better controlled. AR has a significant negative impact on patients’ activities of daily living. Evidence suggests that a substantial number of AR sufferers did not receive medical care for their condition in the past year and/or have not been diagnosed with their condition. Although INCS are the most commonly prescribed AR treatment, less than half of patients are fully satisfied with their INCS. The majority of patients perceive that INCS lose effectiveness over a 24-hour period. The most common reasons for patients to discontinue treatment relate to lack of long-lasting symptom relief rather than side effects. Asthma, sinusitis, nasal polyposis, and sleep apnea are common comorbid conditions and represent an often unrecognized portion of the total burden of disease. Major unmet needs of physicians and patients in AR management include the needs for better therapies and better education.

## Abbreviations

AR: Allergic rhinitis; INCS: Intranasal corticosteroids; OTC: Over-the-counter; MRIA: Marketing Research and Intelligence Association; CSACI: Canadian Society of Allergy and Clinical Immunology; ARIA: Allergic Rhinitis and its Impact on Asthma Workshop Group; AAAAI: American Academy of Allergy Asthma and Immunology.

## Competing interests

Keith: Consultant/Advisory Board for: GlaxoSmithKline, Merck Frosst, Nycomed, Talecris, CSL Behring. Grant/Research support from: GlaxoSmithKline, Nycomed, Allergy Therapeutics, Merck Frosst. Honoraria for lectures from: GlaxoSmithKline, Astra, Merck Frosst, Nycomed, CSL Behring. Waserman: GlaxoSmithKline, Astra Zeneca, Merck Frosst, Nycomed, King Pharma, Paladin Labs, Novartis. Desrosiers: Speakers bureau, advisory boards, consultant: Aventis, GlaxoSmithKline, Schering-Plough, MedtronicXomed, Bayer. Research funding: Fondation Antoine Turmel: Philanthropic organisation. Laister: Currently employed at Nycomed: A Takeda Company in the position of Manager, Medical Liaison-Ontario. Schellenberg: Speakers bureau, advisory boards, consultant: GlaxoSmithKline, Merck Frosst, Novartis, Talecris, Bayer Biologics, and CSL Behring.

## Authors’ contributions

All authors (PKK, MD, TL, ERS, SW) made substantial contributions to the study design and development of the final surveys as well as the analysis and interpretation of the data and the drafting and revision of the manuscript. All authors read and approved the final manuscript.
